# Air Pollution and Its Impact on Health and Performance in Football Players

**DOI:** 10.3390/sports13060170

**Published:** 2025-05-30

**Authors:** George John, Ekaterina A. Semenova, Dana Amr Mohamed, Tiffany Georges Abi Antoun, Rinat A. Yusupov, Ildus I. Ahmetov

**Affiliations:** 1 Transform Specialist Medical Centre, Dubai 119190, United Arab Emirates; drjohnsportsfit@hotmail.com (G.J.); dana.amr2001@gmail.com (D.A.M.); tiffanyabiantoun@hotmail.com (T.G.A.A.); 2 Research Institute of Physical Culture and Sport, Volga Region State University of Physical Culture, Sport and Tourism, 420138 Kazan, Russia; 3 Department of Physical Culture and Sport, Kazan National Research Technical University Named After A.N. Tupolev-KAI, 420111 Kazan, Russia; 4 Laboratory of Genetics of Aging and Longevity, Kazan State Medical University, 420012 Kazan, Russia; 5 Research Institute for Sport and Exercise Sciences, Liverpool John Moores University, Liverpool L3 5AF, UK

**Keywords:** athletes, footballers, soccer, exercise, DNA, genotype, polymorphism, pollutants, mendelian randomization

## Abstract

Air pollution is an escalating global concern with significant implications for human health and athletic performance. This narrative review synthesizes and critically compares the current literature on the impact of air pollution on health and football performance, elucidates the physiological mechanisms involved, and evaluates available mitigation strategies. Comparative studies consistently demonstrate that football players—who frequently engage in high-intensity outdoor exercise—are particularly susceptible to the harmful effects of airborne pollutants such as particulate matter (PM), volatile organic compounds (VOCs), nitrogen dioxide (NO_2_), ozone (O_3_), and carbon monoxide (CO). These pollutants bypass natural respiratory defenses due to increased pulmonary ventilation during exercise, reaching deeper lung regions and triggering oxidative stress, inflammation, and impaired lung function. Evidence across studies indicates that poor air quality is associated with decreased football performance, including reduced distance covered, fewer high-intensity efforts, elevated physiological strain, and diminished training adaptation. Long-term exposure exacerbates respiratory conditions, suppresses immune function, and heightens the risk of illness and injury. Furthermore, comparative genetic research highlights inter-individual variability in pollution sensitivity, with specific gene variants conferring either increased vulnerability or resilience to adverse effects. This review also explores practical and emerging mitigation strategies—such as timing training to avoid peak pollution, utilizing air quality monitoring and antioxidant-rich diets, and promoting sustainable infrastructure—to safeguard athlete health and optimize performance. Novel approaches including respiratory training, anti-smog masks, indoor sessions, and personalized recovery protocols offer additional protection and recovery support.

## 1. Introduction

Air pollution poses a growing global threat with profound implications for human health [1]. Comprising a complex mixture of pollutants, including particulate matter (PM)—categorized by size as PM_10_ (coarse particles with a diameter of 10 μm or less), PM_2.5_ (fine particles under 2.5 μm, linked to combustion), and ultrafine particles (PM_0.1_, smaller than 0.1 μm)—as well as nitrogen oxides (NO_x_), including nitrogen monoxide (NO), nitrogen dioxide (NO_2_), and nitrous oxide (N_2_O), along with ozone (O_3_), sulfur dioxide (SO_2_), carbon monoxide (CO), and volatile organic compounds (VOCs), ambient pollution originates from a range of sources. These include vehicle emissions, industrial activity, biomass burning, and natural sources like dust and wildfires. These pollutants are now well recognized for their capacity to harm multiple biological systems, with both acute and chronic exposure linked to increased morbidity and mortality worldwide [2].

The respiratory system is the primary route of exposure to airborne pollutants. Inhaled particles—particularly those ≤ 2.5 microns in diameter (PM_2.5_)—can evade upper airway defenses and reach the alveoli, where they trigger oxidative stress, inflammation, and structural damage to lung tissue [3]. These responses compromise pulmonary function and can exacerbate respiratory diseases such as asthma and chronic obstructive pulmonary disease (COPD) [4]. In healthy individuals, short-term pollution exposure reduces forced expiratory volume (FEV_1_), forced vital capacity (FVC), and peak expiratory flow (PEF) [5]. Chronic exposure is associated with impaired lung development in children [6], accelerated pulmonary aging in adults [7], and an increased risk of lung cancer [8].

The consequences of air pollution are not limited to the lungs. Systemic inflammation and oxidative stress contribute to endothelial dysfunction, hypertension, and cardiovascular disease [9], as well as breast and prostate cancer [10]. Emerging evidence also highlights the neurotoxic effects of pollutants [11], with associations observed between chronic air pollution exposure and cognitive decline [12], schizophrenia spectrum disorder, depression, and anxiety disorders [13]. The underlying mechanisms likely involve the direct transport of ultrafine particles to the brain via the olfactory nerve or systemic inflammatory pathways, affecting the central nervous system [14]. These health burdens collectively impair quality of life and subjective wellbeing. Symptoms such as fatigue, headaches, sleep disturbances, and mood changes are commonly reported among those exposed to polluted environments [15,16].

Air pollution presents distinct challenges for physically active individuals. Exercise increases minute ventilation and the depth of breathing, resulting in a greater inhalation of pollutants and deeper penetration into the lungs [17]. Outdoor athletes—and in some cases, indoor athletes such as ice hockey players and swimmers—are particularly vulnerable, especially when engaged in prolonged or intense activity in polluted environments [18,19,20]. The cumulative effects of pollution on such populations can be especially detrimental due to the combination of repeated exposure and heightened respiratory demand [21]. Athletes often train and compete in urban environments where pollution levels are highest and may be exposed during early morning or late afternoon sessions when certain pollutants like ground-level O_3_ peak [22]. Even short-term spikes in air pollution have been shown to impair performance, increase respiratory symptoms, and delay recovery in athletes [23,24].

Football players are an at-risk group due to the high-intensity, intermittent nature of the sport, and the frequent conduct of matches and training outdoors [25]. This narrative review aims to summarize the current evidence on the impact of air pollution on health and football performance, elucidate potential mechanisms underlying these effects, and identify effective mitigation strategies to protect athlete health and performance.

## 2. Literature Search

To identify relevant research and review articles, a comprehensive literature search was conducted using PubMed, Scopus, SportDiscus, and Google Scholar, covering the period from January 2000 to April 2025. The search focused on studies examining the effects of air pollution on athletic performance, with a particular emphasis on football (soccer) players. Boolean operators (“AND”, “OR”) and truncation (“*”) were used to enhance the breadth and precision of the search. In PubMed, MeSH terms such as “Air pollution”, “Exercise”, “Athletic performance”, “Football”, “Soccer”, “Particulate matter”, “Volatile organic compounds“, “Nitrogen dioxide”, “Ozone”, and “Carbon monoxide” were combined with free-text terms including “Performance”, “Recovery”, “Training adaptation”, “Cardiovascular stress”, “VO_2_max“, “Respiratory function”, “Oxidative stress”, “Genetic susceptibility”, “Polymorphism”, “GWEIS”, “Mendelian randomization”, and “Mitigation strategies”.

Regarding inclusion criteria, publications were included if they (a) reported data on the effects of air pollution on health, performance, or recovery in football players or physically active individuals; (b) investigated the physiological, genetic, or training-related impact of air pollutants (e.g., PM, NO_2_, O_3_, CO) during exercise; (c) discussed mitigation strategies or interventions to reduce pollution-related harm in athletes; (d) were published in peer-reviewed journals and written in English.

Regarding exclusion criteria, studies were excluded if they (a) did not assess the impact of air pollution on physical performance, recovery, or health outcomes related to sport or exercise; (b) were not peer-reviewed (e.g., conference abstracts, editorials, grey literature); (c) were not available in English.

Studies were not excluded based on athlete level (e.g., elite vs. recreational) or age group, allowing for a comprehensive overview across populations. Both acute and chronic effects of pollution exposure were considered. To ensure thorough coverage, backward citation tracking (snowballing) was performed by reviewing reference lists of relevant articles, particularly systematic reviews and meta-analyses. This iterative process helped identify additional studies that met the inclusion criteria and enriched the narrative synthesis.

## 3. Literature Review

### 3.1. Mechanisms of Air Pollution Impact on Human Health and Performance

The health effects of air pollution involve complex and interconnected biological pathways that are not yet fully understood. A central mechanism is mitochondrial damage caused by particulate matter (PM), especially ultrafine particles [26]. These tiny particles penetrate cells and accumulate in mitochondria, where they disrupt the electron transport chain (ETC). This disruption leads to the excessive production of reactive oxygen species (ROS), including superoxide radicals (O_2_^•−^) and hydrogen peroxide (H_2_O_2_). The resulting oxidative stress damages mitochondrial DNA (mtDNA) and triggers the release of cytochrome C, ultimately initiating apoptotic cell death [26]. In addition, certain transition metals (e.g., iron and copper) present in PM further exacerbate oxidative stress through Fenton reactions, which generate highly reactive hydroxyl radicals (^•^OH). These radicals amplify cellular damage by oxidizing lipids, proteins, and DNA, contributing to inflammation and tissue injury [27].

Additional oxidative damage arises from ozone (O_3_) and ROS generated in response to PM exposure [26,28]. Oxidative stress triggered by environmental exposures can activate downstream inflammatory signaling pathways, including mitogen-activated protein kinase (MAPK), nuclear factor-kappa B (NF-κB), and activator protein 1 (AP-1). This activation promotes cytokine production, immune cell activation, and the onset of inflammation [29,30].

During high-intensity activity, nasal filtration is often bypassed through oral breathing, further increasing pollutant exposure [17]. Acute effects include elevated perceived exertion, reduced oxygen uptake, and impaired aerobic performance [31,32]. These responses are driven by pulmonary irritation, airway resistance, and altered cardiovascular function [24]. Chronic exposure can attenuate training-induced physiological adaptations, diminish VO_2_max, and compromise recovery [33]. Oxidative stress and systemic inflammation may interfere with mitochondrial efficiency, muscle repair, and overall energy metabolism [34]. A detailed summary of these pathways, including their cellular and systemic interactions, is presented in Table 1, which synthesizes the current evidence on how air pollution disrupts physiological function and limits exercise potential.

Air pollution has multifaceted effects on human health that initiate molecular-level changes—such as alterations in the epigenome, transcriptome, proteome, and metabolome—that cascade through cellular, tissue, organ, and systemic levels. These mechanisms collectively contribute to impaired performance and the development and progression of various diseases. Understanding how air pollution affects human health is crucial for developing effective mitigation strategies.

Exposure to air pollution has been increasingly linked to alterations in epigenetic markers, particularly DNA methylation (DNAm); however, the precise mechanisms and long-term health implications remain incompletely understood [29]. DNAm, the addition of methyl groups to cytosine residues—primarily at CpG dinucleotides—plays a critical role in gene regulation. Air pollution appears to disrupt these patterns, frequently leading to global hypomethylation, though site-specific effects and their functional consequences require further investigation [29]. Air pollution-associated DNAm changes often involve immune-related genes (e.g., *IL6*, *ICAM1*), implicating inflammatory pathways [40].

Notably, exposure duration and pollutant concentration influence the magnitude of effects, with longer averaging periods and higher pollution levels generally yielding stronger associations [41]. More specifically, Panni et al. [41] found that short- to mid-term exposure to PM_2.5_ is associated with significant changes in DNA methylation, with the strongest effects observed over 28-day exposure windows. Identified genes (*NSMAF*, *MSGN1*, *NXN*) are linked to inflammatory responses, oxidative stress, and metabolic regulation, suggesting potential mechanisms for PM_2.5_-related health effects. Most CpGs showed increased methylation with higher PM_2.5_ exposure, including cg19963313 (*NSMAF*), cg23276912 (*C1orf212*), cg03455255 (*TSPYL6/ACYP2*), cg11046593 (*MSGN1*), cg04423572 (*ACVR2B-AS1*), cg19215199 (*ZMIZ1*), cg13527922 (*F2*), and cg26003785 (*NXN*), though three sites (cg16308101 in *SERBP1*; cg13169286 in an intergenic region, chr10; and cg20680669 in *MN1*) exhibited decreased methylation, indicating complex and possibly site-specific epigenetic responses that may reflect differential regulatory mechanisms depending on genomic context and exposure duration. The findings highlight the time-dependent nature of air pollution’s epigenetic impact, with some methylation changes appearing only after prolonged exposure.

Mechanistically, oxidative stress may deplete methyl donors or modulate enzymes like DNA methyltransferases (DNMTs) and Ten-Eleven Translocation enzymes (TETs), skewing methylation balance. Interventions such as B-vitamin supplementation show promise in mitigating these effects, likely by supporting one-carbon metabolism [42].

Transcriptomics, the comprehensive analysis of RNA transcripts in a given sample, offers a powerful approach for investigating how air pollutants influence gene expression and related biological pathways [43,44]. By profiling gene expression changes following exposure to environmental toxins, researchers can elucidate the molecular mechanisms underlying respiratory and systemic diseases linked to air pollution. High-throughput technologies such as microarrays and RNA sequencing (RNA-Seq) have enabled the broad-scale investigation of gene expression changes in response to air pollution. These approaches have been applied across various biological models, including human respiratory epithelial cell lines, rodent lungs, and ex vivo lung samples [43]. For example, a recent study identified that exposure to PM_2.5_ in children is associated with altered gene expression, particularly suppressing interferon-related and microbial infection response pathways, as revealed through gene set enrichment analysis in two independent cohorts [44]. Exposure to ambient particulate matter in ovalbumin-sensitized asthmatic mice triggered eosinophilic and neutrophilic airway inflammation and heightened bronchial hyper-responsiveness. Microarray analysis revealed 436 differentially expressed genes by day 4, implicating pathways linked to innate immunity, allergic inflammation, chemotaxis, complement activation, and host defense, reinforcing the pro-inflammatory and asthma-aggravating effects of particulate matter [45]. The study by Wen et al. [46] employed a transcriptome-wide association study approach to uncover shared genetic mechanisms between air pollutant exposure and autoimmune diseases. The analysis identified 71, 68, 70, and 40 overlapping genes associated with NO_2_, NO_x_, PM_2.5_, and PM_10_ exposure, respectively, showing consistent expression patterns in autoimmune conditions. Pathway enrichment revealed the significant involvement of these genes in metabolic processes, immune regulation, and epigenetic modifications. Notably, 11 hub genes—*BEND3*, *PPA2*, *PSMG2*, *RNF40*, *ZMYM1*, *ZNF780A*, *SFR1*, *NDST2*, *SLC35A1*, *OLIG1*, and *PI4KB*—emerged as key molecular links, providing mechanistic insights into how air pollutants may contribute to autoimmune pathogenesis [46].

Growing evidence indicates that air pollution induces significant changes in the human proteome, influencing inflammatory, oxidative stress, and immune response pathways [47]. For example, the human exposure study revealed that diesel exhaust, a model of traffic-related air pollution (TRAP), significantly altered 342 plasma proteins—particularly fractalkine (CX3CL1), apolipoproteins (APOB, APOM), IL18R1, MIP-3 (CCL23), and MMP-12—implicating inflammatory pathways (IL-6/IL-18 signaling) and lipid metabolism (APOB-mediated atherosclerosis) in TRAP-induced cardiovascular disease [48]. Another large-scale proteomic analysis linked 29 environmental exposures to >6500 circulating proteins, revealing key signatures involving DNA damage (TP53, BRCA1), fibrosis (TGF-β, COL1A1), inflammation (IL-6, TNF-α), and mitochondrial function (PPARGC1A) that predicted cardiometabolic/respiratory disease and mortality [49]. Finally, the proteomic study of 50,553 participants revealed that air pollution interacts with key plasma proteins—including CDHR5, TNFRSF13C, ICAM5, and HSD11B1—to modulate depression risk through neuroinflammatory, endocrine (steroid biosynthesis), and metabolic (lipid digestion) pathways, with drug–protein interactions implicating antipsychotics (risperidone) and hormones (progesterone) as potential modifiers of pollution-induced depression [50].

Air pollution exposure also induces significant perturbations in the human metabolome, affecting stress hormones, energy metabolism, and inflammatory pathways. Li et al. [51] revealed that short-term PM_2.5_ exposure elevates stress-related metabolites, including cortisol, cortisone, epinephrine, and norepinephrine, alongside disruptions in glucose, amino acids, fatty acids, and lipids, suggesting activation of the hypothalamus–pituitary–adrenal and sympathetic–adrenal–medullary axes. Liang et al. [52] identified 95 metabolites associated with long-term air pollution exposure, including taurine, creatinine, and sebacate, enriched in pathways linked to oxidative stress, inflammation, and xenobiotic metabolism. Zhuo et al. [53] further demonstrated that air pollution-related metabolomic signatures—comprising 103 metabolites for PM_2.5_ and 85 for NO_x_—were associated with increased chronic respiratory disease risk, mediated by inflammatory and erythrocyte-related markers, and implicated perturbed energy metabolism (e.g., glycolysis, TCA cycle) and amino acid metabolism (e.g., branched-chain amino acids).

Collectively, air pollution exerts complex, multi-layered effects disrupting molecular homeostasis across epigenetic, transcriptomic, proteomic, and metabolomic levels. These alterations propagate oxidative stress, inflammation, and mitochondrial dysfunction, contributing to cardiovascular, respiratory, and neuropsychiatric diseases.

### 3.2. Impact of Air Pollution on Health and Exercise-Related Traits

Air pollution is a leading environmental risk factor contributing to global morbidity and mortality. Exposure to ambient air pollutants has been consistently associated with adverse health outcomes, including cardiovascular disease, respiratory illness, metabolic dysfunction, neurodegeneration, and reduced life expectancy [54,55]. Although epidemiological studies have identified robust associations between air pollution exposure and a wide array of health-related traits, establishing causality remains a significant methodological challenge [2]. A major difficulty in identifying causal effects stems from the presence of residual confounding and measurement error in observational research. Socioeconomic status, behavioral patterns, comorbidities, and spatial misclassification of pollution exposure are often inadequately controlled, limiting the interpretability of associations. Moreover, ethical and logistical constraints make randomized controlled trials of air pollution exposure infeasible, further restricting the potential to derive causal inferences using conventional designs.

To overcome these barriers, Mendelian randomization (MR) has emerged as a powerful tool to strengthen causal inference in environmental epidemiology [56]. MR is a method that uses genetic variants as instrumental variables (IVs) to estimate the causal effect of an exposure (e.g., air pollution or its biological mediators) on a disease outcome or exercise-related trait [57]. The rationale is grounded in the random assortment of alleles at conception, which mimics the randomization process of a clinical trial and helps to mitigate confounding and reverse causation.

In a typical MR study, single nucleotide polymorphisms (SNPs) that are robustly associated with an exposure of interest are selected as proxies. These SNPs must satisfy the following three core assumptions: (1) they are associated with the exposure (relevance), (2) they are not associated with confounders of the exposure–outcome relationship (independence), and (3) they influence the outcome only through exposure (exclusion restriction). MR estimates are often derived using two-sample MR approaches, where summary statistics from genome-wide association studies (GWAS) of the exposure and outcome are analyzed independently, enhancing statistical power and facilitating large-scale analyses [56,58].

Mendelian randomization studies provide robust evidence that air pollution—particularly PM_2.5_, PM_10_, NO_2_, and NO_x_—has widespread causal effects on health- and exercise-related traits, as presented in Table 2. Exercise-related outcomes such as reduced appendicular lean mass, walking pace, grip strength, vitamin D levels, and diminished lung function (FEV_1_, FVC) suggest compromised musculoskeletal and cardiorespiratory capacity. Air pollutants are also strongly linked to cardiovascular diseases, including myocardial infarction, heart failure, stroke, hypertension, and atrial fibrillation. Respiratory conditions such as asthma, COPD, and pneumonia are consistently associated with particulate and gaseous pollutants. Furthermore, causal relationships have been identified with metabolic disorders (type 2 diabetes, obesity, hypercholesterolemia), neurological and psychiatric conditions (Alzheimer’s disease, Parkinson’s disease, depression, anxiety, schizophrenia), cancers (breast, colorectal, endometrial, head/neck), autoimmune diseases (e.g., rheumatoid arthritis, lupus), and oral health issues (periodontitis, gingivitis). Collectively, these findings highlight the systemic burden of air pollution and its detrimental impact on chronic disease risk, physical performance, and overall health.

As the availability of GWAS data continues to expand, and as polygenic scores for pollution exposure and intermediate phenotypes are refined, MR is poised to play an increasingly central role in environmental health research. Future work should integrate MR with other causal inference frameworks and high-resolution exposure mapping to better inform policy interventions and public health strategies aimed at mitigating the health burden of air pollution.

### 3.3. Air Pollution and Its Impact on Health, Performance, and Recovery in Football Players

Outdoor exercise, particularly in sports like football, exposes athletes to air pollution, which can significantly affect their health and performance [92]. For athletes, especially football players who routinely perform high-intensity exercise outdoors, the implications are more acute. Elevated ventilation rates during exertion increase the volume of pollutants inhaled, thereby intensifying the physiological burden [17]. A growing body of evidence indicates that air pollution negatively impacts athletic performance, recovery, and overall well-being, as discussed in the following sections.

A study by Boussetta et al. [93] evaluated the effect of polluted versus clean air on diurnal variations in anaerobic performance, cardiovascular function, and blood parameters in soccer players following the Yo-Yo Intermittent Recovery Test Level-1 (YYIRT1). Eleven healthy soccer players were assessed in both polluted and non-polluted environments at two times of day (08:00 and 18:00). Performance, cardiovascular indices, and hematological markers were significantly impaired in polluted settings. Specifically, there were marked decreases in agility, maximal oxygen uptake (VO_2_max), red blood cells, hemoglobin, pH, and bicarbonate levels in the polluted environment. Concurrently, heart rate, systolic blood pressure, white blood cells, neutrophils, lymphocytes, and carbon dioxide pressure were significantly elevated, indicating heightened physiological stress. These effects were particularly pronounced during evening sessions, likely due to circadian fluctuations in cardiovascular and metabolic responses. The study concluded that polluted environments impair performance and recovery, with evening sessions posing an increased risk to health and performance stability [93].

Building on these findings, Boussetta et al. [94] investigated whether dietary antioxidants could mitigate pollution-induced damage. The study examined the effects of red orange juice supplementation on performance, cardiovascular strain, muscle damage, and oxidative stress following the YYIRT1 test under polluted conditions. Eleven soccer players consumed either red orange juice or a placebo prior to exercise. Although pollution still negatively influenced VO_2_max, heart rate, and systolic blood pressure under both conditions, red orange juice supplementation significantly reduced markers of muscle damage (creatine kinase) and oxidative stress (malondialdehyde). These results suggest that natural antioxidants can offer partial protection against pollution-induced physiological disturbances, supporting dietary strategies as a non-invasive intervention to improve recovery and resilience under polluted conditions [94].

The detrimental impact of air pollution has also been documented in large-scale observational studies of elite footballers. Zacharko et al. [95] analyzed 8927 match observations from 461 players in the German Bundesliga and found that both total distance covered and high-intensity efforts were significantly reduced with increasing concentrations of O_3_, particulate matter, and nitrogen dioxide. This trend was consistent irrespective of the pollutant type, suggesting that even modest air pollution exposure can hinder performance across a spectrum of aerobic and anaerobic tasks. Notably, performance impairments occurred even at levels considered within acceptable environmental thresholds, emphasizing that current regulatory standards may not adequately protect athletes [95].

A follow-up study by Zacharko et al. [96] focused on the influence of PM_10_ levels on players in the Polish Ekstraklasa league. Data from 4294 match observations revealed that players performed better in northern regions of Poland, where air quality was significantly better. Conversely, in the central and southern regions, where PM_10_ levels were higher, both total distance and high-speed running declined. This geographic variation provided natural quasi-experimental evidence of the negative effect of air pollution on physical output, suggesting that even short-term exposure can reduce athletic efficiency and potentially influence match outcomes [96].

Air pollution not only affects professional athletes but also poses risks to developing players. In a longitudinal study, Beavan et al. [97] tracked external load, internal load, and wellness indicators in an elite adolescent soccer team across 26 matches and 197 training sessions. Using pollution data synchronized with each session, the study found that higher concentrations of PM_10_ and O_3_ were significantly associated with reduced total distance covered, increased perceived exertion, and elevated average heart rate. Furthermore, elevated pollutant doses were linked to reduced wellness scores the following day, reflecting compromised recovery. These findings underscore the cumulative toll of pollution on adolescent athletes and reinforce the importance of reducing exposure during critical periods of physiological development [97].

Rundell et al. [98] conducted an environmental assessment of particulate pollution on school and university athletic fields situated near high-traffic roadways. Ultrafine particulate matter (PM_1_) was measured serially over 17 days at four elementary school playgrounds and over 62 days at a university football field. The study revealed that fields located closer to highways exhibited markedly elevated PM_1_ concentrations compared to those situated further away. At the university football field, particle levels were highest near the adjacent roadway and decreased logarithmically with distance, following a second-order decay function. O_3_ concentrations also peaked during warmer afternoon periods, compounding exposure risks. While the study did not directly assess athletic performance or health outcomes, it provided critical environmental exposure data, highlighting potential long-term respiratory and cardiovascular risks for children and young adults who regularly engage in physical activity—such as football training and matches—under polluted conditions. The authors underscored the need for further research to establish exposure thresholds and elucidate the mechanistic effects of chronic particulate inhalation on developing athletes.

While previous studies primarily focused on physical output, Beavan et al. [25] expanded the scope by incorporating technical and cognitive performance in a sample of 799 professional football players. The researchers found that higher concentrations of PM_10_ and O_3_ led to slower sprint and change-of-direction times, as well as decreased technical accuracy. Additionally, nitrogen dioxide exposure negatively affected executive functions, such as attention and response inhibition. This study offered a holistic perspective, highlighting that air pollution compromises multiple performance domains that are essential for football, including decision-making, reaction time, and skill execution. This multidimensional impairment illustrates the broad spectrum of pollution’s impact on elite athletic performance [25].

Zhang et al. [99] further emphasized the cognitive consequences of air pollution using an instrumental variable approach to assess performance in Chinese footballers. Utilizing thermal inversion as an exogenous source of variation in pollution levels, the authors demonstrated that a standard deviation increase of one in air quality index resulted in a 2.5% decline in the number of successful passes and a 5.1% increase in fouls committed. These findings support the hypothesis that pollution-related performance decrements are especially pronounced in cognitively demanding aspects of the game. Interestingly, no significant impact was found on physical parameters such as running distance, reinforcing the view that air pollution primarily compromises neuromotor coordination, concentration, and tactical decision-making [99].

**Figure 1:**
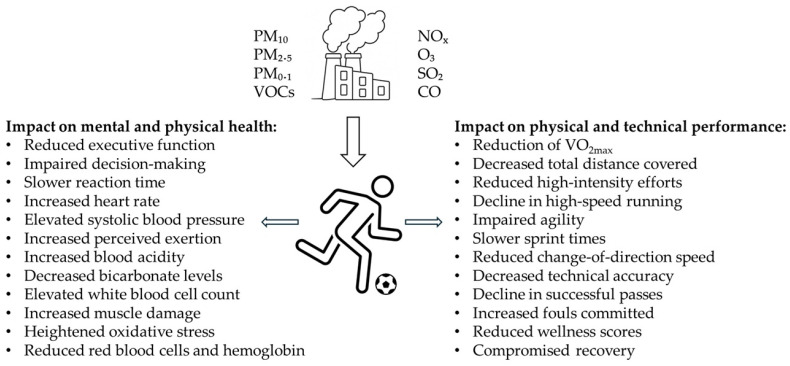
Impact of air pollution on football players: effects on health, performance, and recovery.

Figure 1 summarizes the effects of air pollution on the health, performance, and recovery of football players based on the existing literature. It highlights key impacts across multiple domains, including reduced physical and technical performance, impaired cognitive function, increased cardiovascular stress, compromised recovery, altered hematological markers, and elevated muscle damage and oxidative stress, illustrating the significant challenges posed by polluted environments for athletes.

### 3.4. Sex- and Genotype-Dependent Variability in the Health Effects of Air Pollution

Inter-individual variability in the health effects of air pollution is shaped by both biological sex and genetic background, which influence physiological responses via distinct but interacting mechanisms [100,101]. Epidemiological evidence shows sex-based differences in susceptibility. Women tend to have narrower airways and heightened airway reactivity, which may explain their stronger respiratory responses to air pollution, particularly in older age groups [100,102]. For example, the ARIC study reported greater declines in FEV_1_ and FVC in women living near major roads [103], and a multi-city U.S. study found stronger associations between air pollution and respiratory mortality in women [104].

In children, susceptibility patterns vary by age and developmental stage. Boys may be more vulnerable in early life due to smaller airway size relative to lung volume [105,106], while post-pubertal girls often show stronger effects, possibly due to hormonal changes or activity patterns [107,108]. Sex-specific DNA methylation and gene expression further modulate vulnerability. These findings underscore the need to include sex as a biological variable in research, especially since female football players may face both greater pollution-related health risks and higher injury rates [109,110].

Genetic factors also play a major role in individual susceptibility to air pollution. Variants in genes related to antioxidant defense (e.g., *GSTM1*, *GSTP1*), inflammation (e.g., *TNF*, *IL6*), xenobiotic metabolism (e.g., *CYP1A1*, *EPHX1*), and DNA repair (e.g., *XRCC1*) have been linked to differential responses [101]. For instance, individuals with null genotypes of *GSTM1* or *GSTT1* may have a reduced ability to detoxify reactive oxygen species, increasing oxidative stress and inflammation [111]. Understanding these genetic influences is critical for identifying at-risk athletes and designing personalized strategies to reduce pollution-related health and performance risks.

Recent advances in genome-wide approaches have enabled large-scale assessments of gene–environment interactions across diverse health traits. Genome-wide gene–environment interaction studies (GWEIS) extend traditional GWAS by incorporating interaction terms to identify loci whose effects are modified by environmental exposures such as air pollution [112]. These studies offer valuable insights into biological pathways underlying complex traits and help explain individual differences in susceptibility to environmental risks.

A notable GWEIS on air pollution and lung function by Imboden et al. [113] analyzed how long-term exposure to PM_10_ interacts with genetic variation to affect lung function decline. Using data from the SAPALDIA cohort, they conducted a two-stage genome-wide interaction analysis, focusing on annual decline in forced mid-expiratory flow (FEF25–75%), a marker closely correlated with VO_2_max [114]. The strongest interaction was identified for variant rs2325934 (*p* = 8.8 × 10^−10^) in the *CDH13* gene, which encodes a protein linked to adiponectin, an anti-inflammatory adipokine involved in metabolic and inflammatory processes.

Kim et al. [115] conducted one of the first GWEIS in an Asian population, examining how PM_10_ exposure interacts with genetic variants to affect lung function in 1826 Korean adult men. A significant interaction was found between PM_10_ and rs12312730 in the *BICD1* gene (involved in intracellular transport and neuronal development), influencing forced vital capacity (FVC), which is a key pulmonary metric relevant to football performance [116]. Additionally, two SNPs near the *IL1RN*–*IL1F10* locus (rs6743376 and rs17042888), involved in immune and inflammatory regulation, were associated with reduced FVC under higher PM_10_ exposure. These findings were replicated in an independent cohort of 892 Korean adults, supporting a genetic basis for pollution-related lung function decline [115]. In a follow-up GWEIS, Kim et al. [117] investigated how long-term PM_10_ exposure affects blood pressure in 1868 Korean adults. Stratified by exposure level, the study identified a significant interaction between PM_10_ and rs12914147, located near the *NR2F2* gene, which is involved in inflammation and cardiovascular development. This interaction was confirmed in both discovery and replication analyses [117].

Ierodiakonou et al. [118] conducted a GWEIS to examine how genetic variation modulates the longitudinal impact of NO_2_ on post-bronchodilator FEV_1_—a predictor of VO₂max [119]—in asthmatic children. In Caucasians, significant interactions were found for SNPs rs13090972 and rs958144 near *EPHA3* (linked to immune and developmental processes) and rs7041938 in *TXNDC8* (involved in redox regulation and oxidative stress) [118].

Melbourne et al. [120] analyzed air pollution–gene interactions in ~300,000 UK Biobank participants, focusing on spirometry traits and exposures to PM_2.5_, PM_10_, and NO_2_. Seven novel genome-wide interaction signals were identified. Notably, rs10841302 near *AEBP2* (involved in epigenetic regulation) showed a significant interaction with PM_2.5_ for the FEV_1_/FVC ratio, indicating increased susceptibility in G allele carriers [120].

Peng et al. [121] explored whether rs2237892 in *KCNQ1* (a gene modulating insulin secretion) modifies the effect of PM exposure on fasting blood glucose (FBG) in a Chinese cohort. Each 10 μg/m^3^ increase in PM_2.5_ or PM_10_ was linked to higher FBG, with CC genotype carriers showing the greatest response, highlighting a gene–environment interaction affecting glycemic regulation [121].

Meng et al. [122] used GWEIS in 104,385 UK Biobank participants to examine the joint influence of air pollution and polygenic risk on depression and anxiety. Individuals with higher genetic risk experienced greater susceptibility to pollutants (NO, NO_2_, PM_2.5_, PMcoarse), with significant loci including *TSN* (rs184699498, rs139212326) and *HSP90AB7P* (rs150987455) modifying depression outcomes under NO_2_ and PM_2.5_ exposure [122].

Ma et al. [123] assessed gene–pollution interactions in COVID-19 outcomes among 458,396 UK Biobank participants. Long-term PM exposure (PM_2.5_, PM_10_) was associated with higher risks of hospitalization and death. Key loci involved in these interactions included SLC6A20 (rs11385942), *LZTFL1* (rs35081325), *DPP9* (rs2109069), and *IFNAR2* (rs2236757), demonstrating the role of gene–environment interplay in COVID-19 severity [123].

Collectively, genome-wide gene–environment interaction studies highlight the critical role of genetic variation in modulating the health effects of air pollution. The implicated genes span pathways related to immune function, oxidative stress, mitochondrial activity, neurodevelopment, and aging. While conducted in general populations, these findings suggest that similar interactions could impact football-relevant traits, including lung function, infection susceptibility, cardiovascular and glucose regulation, and mental health. Integrating genetic screening with pollution exposure data may help identify at-risk players and support personalized strategies for training, recovery, and protection. Moreover, such testing could be employed to assess genetic potential in traits crucial for football success—such as power [124,125,126], strength [127,128], endurance [129,130,131,132], reaction time [133], technical capabilities [134], testosterone levels [135,136], muscle mass [137,138], and resistance to injuries [139,140]—thereby informing personalized training, recovery, and environmental risk mitigation strategies.

### 3.5. Mitigation Strategies for Football Players in Response to Air Pollution

Given the substantial risks to the health and performance of football players, the implementation of targeted mitigation strategies is essential. Emerging evidence from the literature indicates that several effective approaches already exist, while others remain under discussion [141,142].

#### 3.5.1. Temporal and Spatial Avoidance of Pollution Peaks

Mitigation begins with reducing exposure time and avoiding high-exposure locations. Football players are encouraged to schedule training during times of day when pollutant concentrations are at their nadir, typically in the early morning when traffic-related air pollution (TRAP) and O_3_ levels are reduced [142]. This temporal strategy aligns with findings from Reche et al. [143], who reported significant diurnal variations in NO_2_ concentrations during the World Relays in Yokohama, with late-night and early morning hours showing the lowest levels. Spatial avoidance, including the selection of training locations distant from high-traffic areas, is another recommended tactic. Greenways, which are vegetated corridors used for recreation, have been shown to reduce TRAP exposure due to their distance from roadways and natural filtering effects [144].

#### 3.5.2. Monitoring and Forecasting Pollution Levels

Effective mitigation depends on access to accurate and timely air quality data. The adoption of air quality monitoring—via public Air Quality Index (AQI) systems, stadium-based sensors, or personal wearable technologies—is a critical step. Fixed-site monitors often provide generalized data over large areas, which may not reflect individual exposure, particularly in stadium microenvironments or urban exercise routes [141,145]. To bridge this gap, Viana et al. [145] deployed high-resolution sensors in stadia worldwide and demonstrated their utility in discerning pollutant sources (e.g., NO_2_ from traffic, PM_10_ from dust) and identifying optimal periods for scheduling events. The use of wearable sensors, such as silicone wristbands, has also gained traction as a tool to assess individual exposure to polycyclic aromatic hydrocarbons (PAHs) and other organic pollutants [143]. These data enable athletes and coaches to personalize exposure management strategies and foster informed decision-making.

#### 3.5.3. Acclimation Protocols and Pre-Exposure Conditioning

The concept of pollution acclimation remains controversial but is supported by preliminary evidence in relation to O_3_ exposure. Mullins et al. [146] found that athletes who experienced the greatest performance losses due to O_3_ were those least exposed in the preceding week. Despite these findings, controlled exposure protocols cannot be currently recommended due to safety concerns and limited generalizability across pollutants [142]. For other common air pollutants such as PM and NO_2_, no comparable acclimation benefits have been identified [142]. On the contrary, acute exposure to diesel exhaust and black carbon before exercise has consistently been associated with impaired pulmonary and vascular function [147,148,149].

#### 3.5.4. Face Masks and Protective Equipment

The adoption of high-filtration face masks (e.g., N95, FFP2) may reduce inhaled pollutant load during low-intensity activities. However, their utility in high-intensity sport remains limited. Walsh et al. [142] noted that mask use during exercise induces discomfort, elevated thermal perception, and higher rates of perceived exertion. Moreover, scientific studies assessing the efficacy of masks during moderate to vigorous physical activity are scarce. While not suitable for use during competition, short-term mask use during warm-ups, commuting, or travel to polluted venues may offer low-cost, low-risk exposure mitigation.

#### 3.5.5. Antioxidant Supplementation

Air pollutants elicit oxidative stress, prompting interest in antioxidant supplementation as a protective measure. O_3_ exposure generates reactive oxygen species that can impair lung function, but dietary antioxidants may mitigate these effects. Two studies by Grievink et al. [150,151] investigated this in amateur cyclists. In the first study [150], 26 cyclists were split into a control group and a supplementation group receiving 15 mg β-carotene, 75 mg vitamin E, and 650 mg vitamin C daily for three months. Lung function tests showed significant ozone-related declines in FVC, FEV_1_, and PEF in the control group but not in the supplemented group, suggesting a protective effect. The second [151] randomized, placebo-controlled study of 38 cyclists confirmed these findings, with the antioxidant group (100 mg vitamin E, 500 mg vitamin C) showing minimal lung function decline compared to significant reductions in the placebo group at ozone levels averaging 77 µg/m^3^. Additionally, a study by Boussetta et al. [94] explored red orange juice supplementation (ROJS) in 11 soccer players performing exercise in polluted (PA) and non-polluted areas (NPA). Participants consumed 500 mL of ROJS or a placebo before a Yo-Yo Intermittent Recovery Test. ROJS reduced muscle damage (creatine kinase levels) and oxidative stress (malondialdehyde levels) post-exercise in both PA and NPA compared to the placebo, indicating ROJS as a potential strategy to mitigate pollution-related damage. These studies collectively suggest that antioxidant supplementation can protect against pollution-induced impairments in active individuals. While promising, these results need replication in larger cohorts and with pollutant-specific contexts. Nonetheless, according to a recent position statement from CASEM and the Canadian Society for Exercise Physiology (CSEP), it is advisable to supplement with 250–650 mg of vitamin C, 75–100 mg of vitamin E, and 25 mg of β-carotene for a minimum of one week before engaging in physical activity in environments with elevated O_3_ levels [141].

#### 3.5.6. Indoor Air Quality Management

Although much of the focus in air pollution research has been on outdoor environments, indoor air quality (IAQ) is also a critical factor influencing athletic performance, particularly for football players who often train indoors in gyms, recovery areas, or indoor pitches and who occasionally compete in enclosed stadiums. Though often overlooked, IAQ can substantially affect physical and cognitive performance in indoor sports settings. For instance, Jiang et al. [152] demonstrated that higher indoor AQI levels were significantly associated with reduced player efficiency ratings (PER) in over 2500 Chinese Basketball Association games, highlighting the tangible impact of air pollution even in indoor environments. Pollutants such as particulate matter, ozone, and volatile organic compounds can accumulate indoors, often mirroring outdoor pollution levels due to poor ventilation or air infiltration. This is particularly relevant for footballers during high-intensity indoor training sessions, strength and conditioning work, or rehabilitation, where elevated ventilation rates increase susceptibility to airborne pollutants. Therefore, robust IAQ monitoring and control measures—such as high-efficiency mechanical ventilation systems, regular maintenance and cleaning protocols, and controlled occupancy in training facilities—are essential to safeguard the health and performance of athletes [18]. Given the elevated respiratory rates and prolonged exposure during indoor training, maintaining optimal air quality standards within indoor sports environments should be considered a priority for football teams and support staff.

#### 3.5.7. Policy and Infrastructure Interventions

Event-based interventions, such as emission controls during major sporting events, have had mixed success. Wang et al. [153] reported negligible changes in PM_1_ levels despite significant reductions in NO_x_ and SO_2_ during the Youth Olympic Games in Nanjing, highlighting the role of meteorological factors (e.g., wind speed, humidity) in pollution accumulation. Consequently, long-term regional emission reductions are favored over short-term controls. Urban health planning must integrate air quality considerations in sports infrastructure design, using tools such as spatial modeling and pollutant source apportionment [145,154].

Overall, while current mitigation strategies offer valuable protection for football players against air pollution, their effectiveness varies. Continued research, better infrastructure, and stronger policies are essential to fully safeguard athletes’ health and performance in polluted environments. A detailed summary of mitigation strategies for football players against air pollution is provided in Table 3.

## 4. Conclusions

Air pollution presents a growing and often underestimated threat to the health, performance, and recovery of football players. It contributes to physiological stress through mechanisms such as oxidative damage, systemic inflammation, and compromised cardiorespiratory function. This review highlights that exposure to key pollutants—including PM_2.5_, PM_10_, NO_2_, and O_3_—is associated with detrimental effects on aerobic capacity, technical precision, cognitive performance, and recovery, even when pollutant concentrations fall within current regulatory limits. Evidence indicates reductions in VO_2_max, elevated markers of muscle damage, impaired high-intensity performance, and deficits in decision-making and motor coordination under polluted conditions. Individual susceptibility is influenced by sex and genetic background, with females and those carrying certain genetic polymorphisms exhibiting heightened vulnerability.

While these findings underscore the urgency of addressing air pollution in sport, research specifically focusing on football players remains limited. Most existing studies have been conducted in general populations or in other sports, leaving a critical gap in understanding how the unique physical, tactical, and environmental demands of football interact with pollutant exposure. This gap extends to mitigation strategies, many of which are yet to be fully validated or adapted for football-specific contexts. While promising interventions—such as the temporal and spatial avoidance of peak pollution periods, antioxidant supplementation (e.g., vitamins C and E, red orange juice), and enhancements in indoor air quality—have shown potential, most evidence comes from general athletic populations rather than elite football players. Therefore, caution is warranted when extrapolating these findings, highlighting the urgent need for targeted football-specific studies to rigorously assess the efficacy and safety of such strategies in this population.

To translate these findings into practical measures, football clubs and governing bodies should integrate air quality monitoring into training and competition planning. Scheduling training sessions and matches to avoid periods of high pollution can reduce acute physiological stress on players. Investments in infrastructure, such as air filtration and improved ventilation systems in indoor training environments, may further mitigate pollutant exposure. Nutritional strategies incorporating antioxidant supplementation tailored to individual susceptibility profiles could enhance resilience against oxidative damage and inflammation.

At the governance level, football organizations need to develop sport-specific policies that address air pollution risks. This includes establishing threshold air quality indices to guide activity modifications, integrating environmental monitoring data into decision-making, and advocating for broader emission reduction strategies in urban areas near sports facilities. Collaborative frameworks involving health professionals, environmental scientists, and sport administrators are essential to develop sustainable policies that protect player health, enhance performance, and ensure safer sporting environments.

As this is a narrative review aimed at providing a broad and integrative overview, no formal quality assessment tool (e.g., PEDro score) was applied. This approach carries an inherent risk of selection and interpretation bias, and the included studies may vary in methodological quality and generalizability.

Future research should prioritize well-designed studies that investigate the short- and long-term effects of air pollution on football performance and health outcomes. Subsequent investigations should also account for the source of pollutant exposure, as emerging evidence suggests that health risks may vary depending on the origin. For instance, Elser et al. [156] reported that each 1-μg/m^3^ increase in PM_2.5_ from wildfire smoke was associated with an 18% increase in dementia risk, compared to only a 1% increase for PM_2.5_ from non-wildfire sources. High-resolution exposure monitoring, combined with genetic and physiological profiling, may enable the development of personalized interventions. In parallel, policy measures must address the need for sport-specific standards and long-term emission reduction strategies. By expanding the evidence base and refining targeted mitigation approaches, the football community can take decisive steps to protect athletes from the insidious and pervasive effects of air pollution, ultimately supporting safer, healthier, and more sustainable sporting environments.

## Figures and Tables

**Table 1:** Mechanisms linking air pollution to impaired performance.

Mechanism	Biological Processes	Physiological Effects	Impact on Performance	Key Air Pollutants
Pulmonary irritation [35]	Activation of airway sensory nerves (e.g., C-fibers); release of neuropeptides (substance P, CGRP); recruitment of immune cells	Airway inflammation; epithelial damage; mucus production; local edema	Coughing; chest tightness; elevated perceived exertion (RPE)	O_3_, SO_2_, NO_2_, PM_2.5_, PM_10_, formaldehyde, acrolein
Airway resistance [36]	Bronchoconstriction (via vagal reflexes); edema; mucus hypersecretion; particulate obstruction in small airways	Increased airway resistance; reduced airflow; impaired gas exchange	Dyspnea; lower ventilation efficiency; early fatigue	SO_2_, O_3_, NO_2_, PM_2.5_, PM_10_, formaldehyde, toluene
Systemic inflammation [24]	Spillover of cytokines (IL-6, TNF-α); circulating acute-phase proteins (e.g., CRP); endothelial dysfunction	Vascular inflammation; reduced NO-mediated vasodilation; arterial stiffness	Reduced oxygen delivery; decreased cardiovascular efficiency	PM_2.5_, NO_2_, O_3_, benzene, formaldehyde
Autonomic nervous system imbalance [37]	Altered heart rate variability; increased sympathetic tone; decreased vagal activity	Elevated heart rate; vasoconstriction; increased cardiac workload	Elevated RPE; risk of arrhythmias; reduced exercise capacity	PM_2.5_, O_3_, CO, NO_2_, benzene
Oxidative stress [26]	Reactive oxygen species (ROS) production; oxidative damage to membranes, proteins, and DNA; mitochondrial dysfunction	Inflammation and tissue injury; reduced metabolic efficiency	Increased muscle fatigue; impaired recovery	O_3_, PM_2.5_, NO_2_, SO_2_, acrolein, benzene
Blood coagulation changes [38]	Platelet activation; increased fibrinogen; vascular endothelial activation	Hypercoagulability; impaired microcirculation	Reduced muscle perfusion; risk of thrombotic events	PM_2.5_, NO_2_, O_3_, polycyclic aromatic hydrocarbons
Reduced oxygen transport [39]	CO binds hemoglobin (COHb formation); decreased oxygen-binding capacity; hypoxemia	Reduced arterial oxygen content; lower VO_2_ delivery	Decreased VO_2_max; impaired endurance	CO

**Table 2:** Causal effects of air pollution on health and exercise-related traits: a Mendelian randomization analysis.

Pollutants	Affected Traits	References
PM_2.5_	Reduced appendicular lean mass, slow walking pace, and decreased hand grip strength	[59]
PM_10_, PM_2.5–10_, PM_2.5_	Low 25-hydroxyvitamin D (25(OH)D) levels	[60]
PM_2.5_, PM_10_, NO_2_	Reduced forced expiratory volume in one second (FEV1) and reduced forced vital capacity (FVC)	[61]
PM_2.5_, PM_10_, NO_x_	Decreased bone mineral density	[62]
PM_2.5_	Frailty	[63]
PM_2.5_	Asthma	[64]
PM_2.5_	Chronic obstructive pulmonary disease	[65]
PM_10_	Pneumonia and bronchiectasis	[66]
PM_2.5_, PM_2.5–10_, NO_x_	COVID-19 and bacterial pneumonia	[67]
PM_10_, PM_2.5–10_	Type 2 diabetes	[68]
PM_2.5_	Obesity, increased visceral adipose tissue volume, and increased abdominal subcutaneous adipose tissue volume	[69]
PM_2.5_	Gastroesophageal reflux disease	[70]
NO_2_	Hypothyroidism	[46]
NO_2_, NO_x_, PM_2.5_	Diabetic retinopathy and age-related macular degeneration	[71]
PM_2.5_, NO_x_	Osteoporosis	[72]
PM_2.5_	Kidney stones	[73]
PM_2.5_	Hypercholesterolemia	[74]
PM_10_, NO_2_	Myocardial infarction and chronic heart failure	[68]
NO_x_	Stroke	[75]
PM_10_	Hypertension and atrial fibrillation	[76]
PM_2.5_	Heart failure	[76]
NO_2_, NO_x_, PM_2.5_	Anxiety disorders, schizophrenia, and bipolar disorder	[77]
PM_10_	Alzheimer’s disease	[78]
PM_10_	Post-traumatic stress disorder and multiple sclerosis	[79]
NO_2_, NO_x_, PM_2.5_	Major depressive disorder, bipolar disorder, schizophrenia, attention deficit hyperactivity disorder, and autism spectrum disorder	[80]
PM_2.5_, NO_2_	Reduction in cortical surface area	[81]
NO_x_, PM_2.5_	Amyotrophic lateral sclerosis	[82]
NO_2_	Parkinson’s disease	[83]
PM_10_	Acute pharyngitis	[84]
NO_2_	Chronic rhinitis, chronic nasopharyngitis, and chronic pharyngitis	[84]
PM_2.5_, PM_2.5–10_, NO_2_	Oral leukoplakia, gingivitis, periodontitis, pulp diseases, periapical diseases, oral cavity diseases, salivary gland diseases, and jaw diseases	[85]
NO_x_	Rheumatoid arthritis, Sjogren’s syndrome, and systemic lupus erythematosus	[86]
PM_10_	Psoriasis	[86]
PM_2.5_	Ulcerative colitis	[87]
NO_2_, NO_x_, PM_2.5_	Head and neck squamous cell carcinoma	[88]
PM_10_, NO_x_	Breast cancer	[89]
NO_2_	Colorectal cancer	[90]
NO_2_	Endometrial cancer and ovarian cancer	[91]

**Table 3:** Mitigation strategies for football players against air pollution.

Strategy	Description	Key Benefits	Limitations/Challenges
Temporal and spatial avoidance [142,144]	Schedule training in early morning when pollutant levels (e.g., TRAP, O_3_) are low; train in greenways or areas distant from traffic.	Reduces exposure to NO_2_, PM, and O_3_; leverages natural filtration in vegetated areas.	Requires access to pollution data and suitable training locations; may not be feasible in urban settings.
Monitoring and forecasting [145]	Use Air Quality Index (AQI) systems, stadium sensors, or wearable devices (e.g., silicone wristbands) to track pollutant exposure.	Enables personalized exposure management; identifies optimal training times and locations.	Fixed-site monitors may lack precision; wearable tech adoption is limited.
Acclimation protocols [146]	Repeated low-level O_3_ exposure may reduce performance decrements via cardiorespiratory adaptations.	Potential to mitigate O_3_-related performance losses.	Safety concerns; limited evidence for other pollutants (e.g., PM, NO_2_); not widely recommended.
Face masks [155]	Use high-filtration masks (e.g., N95, FFP2) during low-intensity activities, warm-ups, or travel.	Reduces inhaled pollutant load in non-competitive settings.	Uncomfortable during high-intensity exercise; limited evidence for efficacy in sports.
Antioxidant supplementation [94,150,151]	Supplement with vitamins C, E, or red orange juice to counter oxidative stress from pollutants.	May improve lung function and reduce oxidative stress/muscle damage.	Mixed results; requires medical guidance; pollutant-specific benefits unclear.
Indoor air quality (IAQ) management [152]	Monitor and improve IAQ in training facilities using filtration systems.	Protects athletes in indoor arenas; mitigates ambient pollutant infiltration.	Indoor pollutant levels often mirror outdoor conditions; requires infrastructure investment.
Policy and infrastructure interventions [153]	Implement emission controls and integrate air quality into sports facility design.	Long-term regional emission reductions improve air quality; enhances urban planning.	Short-term controls often ineffective due to meteorological factors; requires policy coordination.
